# Association analysis of the gut microbiota in predicting outcomes for patients with acute ischemic stroke and H-type hypertension

**DOI:** 10.3389/fneur.2023.1275460

**Published:** 2023-10-26

**Authors:** Shicheng Yu, Jiaxin Chen, Yiting Zhao, Xiaolan Liao, Qionglei Chen, Huijia Xie, Jiaming Liu, Jing Sun, Shaoce Zhi

**Affiliations:** ^1^Department of Geriatrics, The Second Affiliated Hospital and Yuying Children’s Hospital of Wenzhou Medical University, Wenzhou, Zhejiang, China; ^2^Department of Preventive Medicine, School of Public Health and Management, Wenzhou Medical University, Wenzhou, Zhejiang, China; ^3^Department of Emergency, The First Affiliated Hospital of Wenzhou Medical University, Wenzhou, Zhejiang, China

**Keywords:** H-type hypertension, acute ischemic stroke, gut microbiota, biomarker, 16S rRNA

## Abstract

**Introduction:**

H-type hypertension (HHTN) is a subtype of hypertension that tends to worsen the prognosis of acute ischemic stroke (AIS). Recent studies have highlighted the vital role of gut microbiota in both hypertension and AIS, but there is little available data on the relationship between gut microbiota and the progression of AIS patients with HHTN. In this study, we investigated the microbial signature of AIS patients with HHTN and identified characteristic bacteria as biomarkers for predicting prognosis.

**Methods:**

AIS patients with HHTN (*n* = 150) and without HHTN (*n* = 50) were enrolled. All patients received a modified Rankin Scale (mRS) assessment at 3 months after discharge. Fecal samples were collected from the participants upon admission, including 150 AIS patients with HHTN, 50 AIS patients with non-HHTN, and 90 healthy subjects with HHTN. These samples were analyzed using 16S rRNA sequencing to characterize the bacterial taxa, predict functions, and conduct correlation analysis between specific taxa and clinical features.

**Results:**

Our results showed that the composition of the gut microbiota in HHTN patients differed significantly from that in non-HHTN patients. The abundance of the genera *Bacteroides*, *Escherichia-Shigella*, *Lactobacillus*, *Bifidobacterium*, and *Prevotella* in AIS patients with HHTN was significantly increased compared to AIS patients without HHTN, while the genus *Streptococcus*, *Faecalibacterium*, and *Klebsiella* were significantly decreased. Moreover, *Bacteroides*, *Lactobacillus*, *Bifidobacterium*, and *Klebsiella* in AIS patients with HHTN were more abundant than healthy subjects with HHTN, while *Escherichia-Shigella*, *Blautia*, and *Faecalibacterium* were less abundant. Moreover, the genera *Butyricicoccus*, *Rothia,* and *Family_XIII_UCG-001* were negatively connected with the NIHSS score, and the genera *Butyricicoccus* and *Rothia* were observed to be negatively associated with the mRS score. The genera *Butyricicoccus*, *Romboutsia*, and *Terrisporobacter* were associated with a poor prognosis, whereas the increase in *Butyricimonas* and *Odoribacter* was correlated with good outcomes. Generated by eight genera and clinical indexes, the area under the curve (AUC) value of the receiver operating characteristic (ROC) curve achieved 0.739 to effectively predict the prognosis of AIS patients with HHTN.

**Conclusion:**

These findings revealed the microbial signature of AIS patients with HHTN and further provided potential microbial biomarkers for the clinical diagnosis of AIS patients with HHTN.

## 1. Introduction

Acute ischemic stroke (AIS), one of the leading causes of disability and death worldwide (1, 2), is characterized by high incidence, high recurrence, and high mortality. Hypertension is regarded as a primary risk factor for AIS (1, 3), and most hypertensive patients are accompanied by an increase in serum homocysteine (Hcy) (4, 5), which is defined as H-type hypertension (HHTN), which means a combination of essential hypertension and a high level of Hcy (6). The prevalence of HHTN among hypertension patients varies widely, ranging from 75 to 80.3% (6, 7). Studies have reported that HHTN contributed to more cerebrovascular disease (CVD), such as stroke (8), and ultimately led to higher mortality (9, 10). Compared with simple stroke patients, patients with both HHTN and stroke were reported to have a higher death rate and a greater potential for cognitive disorders (11, 12).

To date, some clinicians primarily rely on risk factors such as hypertension, diabetes, dyslipidemia, and low folic acid (FA) to predict the outcomes of stroke patients (13, 14). The potential clinical risk factors increase the chance of adverse outcomes, and the biomarkers contribute to their prediction and clinical outcomes. Currently, the existing risk factors lack accuracy in evaluating the prognosis for AIS patients with HHTN. Thus, it is urgent to identify more effective biomarkers to predict the adverse outcome of the disease.

Recently, the close relationship between changes in gut microbiota (GM) composition and many diseases has been widely reported (15–17). It was reported that hypertension was closely related to the dramatically decreasing richness of the microbiota (18), and beneficial microbial supplementation was able to prevent the development of hypertension (19). GM also revealed its contribution to regulating hyperhomocysteinemia in Parkinson’s disease (PD) (20), and microbial treatment could mitigate the severity of hyperhomocysteinemia in mouse models (15). A growing body of evidence indicated that GM could be involved in the pathogenesis of neurological disorders (21, 22), such as stroke, and altered GM had been identified as one of the risk factors for stroke (23). Our previous studies revealed that the GM in patients with post-stroke depression (PSD) was characterized by the genera *Streptococcus*, *Akkermansia,* and *Barnesiella*, which were diagnostic microbial biomarkers of PSD (24).

Moreover, another study also demonstrated that post-stroke cognitive impairment (PSCI) patients observed a significant correlation with the abundance of *Enterobacteriaceae*, which was expected to be a biomarker of PSCI patients (25). Our previous study revealed that the abundance of *Gammaproteobacteria* and *Enterobacteriaceae* is negatively correlated with MoCA scores (26). Owing to the different expressions of GM in various apoplectic outcomes (27), some researchers propose using GM as an indicator to assess the prognosis of AIS (28). Moreover, bacteria producing short-chain fatty acids (SCFAs) were found to decrease in stroke patients with hyperlipidemia and the genera, including *Faecalibacterium* and *Butyricicoccus*, were able to facilitate the prognosis and diagnosis of stroke (29). Although diagnostic microbiota has been well studied in neurological diseases, the characteristic taxa in AIS patients and HHTN patients remain unclear.

To determine the microbial characteristics of AIS patients with HHTN, we conducted a prospective study to identify the characteristic taxa and their predictive role in adverse prognosis and further explored the relationship between GM, HHTN, and AIS. This study identified key bacteria that might be involved in the prognosis of AIS patients with HHTN.

## 2. Materials and methods

### 2.1. Participant recruitment

This study involved 200 AIS patients and 90 healthy participants with HHTN who were enrolled at the Second Hospital of Wenzhou Medical University in China from September 2020 to July 2021. The inclusion criteria for AIS patients were as follows: (1) admission within 72 h after the onset of AIS; (2) age ≥ 18 years; and (3) no special dietary habits, such as vegetarianism. The exclusion criteria were as follows: (1) patients who had taken medications such as antibiotics or prebiotics (within 3 months before admission) or medications affecting Hcy levels such as folic acid (FA), vitamins B6, and B12 (VB12) within 3 months; (2) secondary hypertensives caused by sleep apnea syndrome, pheochromocytoma, and so on; and (3) histories of severe gastrointestinal disease, gastrointestinal surgery, malignancy, or pregnancy. A total of 30 AIS patients and three healthy participants were excluded. Finally, we performed GM profiling on fecal samples from 290 subjects, including AIS patients with HHTN (ISHH), non-HHTN AIS patients (NISHH), and HHTN participants without AIS (HH). AIS patients were eligible for the diagnostic criteria of the American Stroke Association (30). ISHH patients were defined as having primary hypertension with systolic blood pressure (SBP) of ≥140 mmHg or diastolic blood pressure (DBP) of ≥90 mmHg, accompanied by Hcy of ≥10 μmol/L. NISHH patients were diagnosed as people without hypertension and Hcy of <10 μmol/L. This study was approved by the medical ethics committee of the Second Affiliated Hospital of Wenzhou Medical University, and informed consent was obtained from each participant.

### 2.2. Demographic and clinical characteristics

Demographic characteristics of each participant were collected by a trained researcher, which included age, gender, educational level, and marital status. Risky lifestyles (smoking and drinking) and previous histories such as diabetes, hyperlipidemia (HL), and CVD were also involved. Blood pressure was measured by a professionally trained nurse. At 0.5 h before blood pressure measurement, patients were advised to avoid strenuous exercise, smoking, coffee, and tea. The measurements were taken three times using an electronic sphygmomanometer, with a minimum rest period of 5 min between each measurement. The mean of these three values was calculated and recorded. Diabetes and hyperlipidemia were diagnosed by professionals in endocrinology. Laboratory indexes involved C-reactive protein (CRP), hypersensitive CRP (Hs-CRP), FA, Vitamin B12 (VB12), D-dimer, troponin, fasting glucose (FBG), glycosylated hemoglobin (HbAlc), triglycerides (TG), total cholesterol (TC), low-density lipoprotein cholesterol (LDL), high-density lipoprotein cholesterol (HDL), aspartate aminotransferase (AST), alanine aminotransferase (ALT), and thyroid stimulating hormone (TSH) were measured. The National Institutes of Health Stroke Scale (NIHSS) was used to assess the degree of neurological damage during hospitalization. The modified Rankin Scale (mRS) was used to assess the post-stroke functional prognosis of each patient 3 months later. In the current study, the mRS score of ≤1 was considered a good prognosis, while the mRS score of >1 was considered a poor prognosis.

### 2.3. Fecal collection and GM analysis

Fecal samples (200 mg) were obtained from patients during hospitalization or outpatient visits. Healthy control group members provided stool samples voluntarily at the health screening center. Each sample was collected and labeled using 2 mL sterile centrifuge tubes, immediately frozen in liquid nitrogen, and stored at −80°C. Total DNA was extracted from the stool samples by the E.Z.N.A.^®^ Soil DNA Kit (Omega Bio-Tek, Norcross, GA, USA), and then, DNA concentration and purity were determined by using a NanoDrop2000 (Thermo Fisher Scientific, Wilmington, USA) for detection. Primers 338F (ACTCCTACGGGAGGCAGCAG) and 806R (GGACTACHVG GGTWTCTAAT) were involved in the PCR amplification of the high mutation region of 16 s rRNA. PCR products were recovered using a 2% agarose gel, sequenced on the Illumina MiSeq platform (Illumina, San Diego, USA), and spliced using FLASH software according to the manufacturer’s protocol.

The Ace and Shannon indices based on the Wilcoxon rank sum test were used to analyze the alpha diversity of the GM. Partial least squares-discriminant analysis (PLS-DA) was used to reflect differences in GM abundance in groups. Moreover, according to the relative abundance of GM, distributions of different groups at various taxonomic levels were determined, including phylum, class, order, family, genus, species, and ASV. In addition, based on the Kruskal–Wallis test, linear discriminant analysis (LDA) effect size (LEfSe) used an LDA score of >2 as the threshold. The Spearman correlation coefficient was used to evaluate the correlation between GM and clinical variables, visualized by heatmap. The receiver operating characteristic (ROC) curve was used to reflect the specificity and sensitivity of microbial characteristics in the prediction of stroke prognosis. All results of GM were performed by GraphPad Prism V.9.0.0 (La Jolla, CA, USA).

### 2.4. Statistical analysis

All data were analyzed by SPSS 26.0 software (SPSS, Chicago, USA). The continuous variables were expressed as the mean ± standard deviation (SD). The other variables were expressed by the median and quartiles. Categorical variables were expressed as numbers and percentages (%). The correlation between HHTN and prognosis was investigated by constructing the multivariate logistic regression models, and all variables were significantly different in the univariate analysis (*p* < 0.05). Relative risks were expressed as odds ratios (OR) with 95% confidence intervals (CI). A *p*-value of <0.05 was considered statistically significant.

## 3. Results

### 3.1. Baseline characteristics of ISHH, NISHH, and HH patients

This study enrolled 200 patients diagnosed with AIS, of whom 150 patients were assigned to the ISHH group and 50 to the NISHH group. The clinical demographics of ISHH and NISHH patients are presented in Table 1. In the ISHH group, the median age was 68 years old, and 70.7% were men, while in the NISHH group, the median age was 65 years old, and 48.0% were men. Diabetes, drinking, SBP, DBP, mean arterial pressure (MAP), mRS score, and laboratory indexes such as Hs-CRP, FA, VB12, uric acid (UA), Hcy, TG, HDL, and TSH showed significant differences between the ISHH and the NISHH groups. The marital status, educational level, CVD, hyperlipemia, smoking, and NIHSS score showed no significant differences between the two groups. Moreover, we recruited 90 healthy participants diagnosed with HHTN who were classified as HH. As shown in Table 1, the median age of HH participants was 71 years old, and 66.7% were men. Drinking, SBP, DBP, MAP, and laboratory indexes such as CRP, Hs-CRP, troponin, FBG, LDL, and FT3 showed differences between the ISHH and HH groups, while marital status, educational level, CVD, diabetes, hyperlipemia, and smoking showed no differences between the two groups. The multivariable logistic regression showed that the HHTN (OR = 2.542, *p* = 0.038) and NIHSS (OR = 1.236, *p* = 0.002) scores were independent risk factors for poor ISHH prognosis, while FA (OR = 0.908, *p* = 0.018) was a protective factor (Table 2).

**Table 1:** Characteristics of the recruited patients.

Variables	ISHH (*n* = 150)	NISHH (*n* = 50)	HH (*n* = 90)	*p*-value (ISHH vs. NISHH)	*p*-value (ISHH vs. HH)
Demographic parameters					
Age (years)	68 (58, 79)	65 (57, 73)	71 (65, 76)	0.143	0.310
Male sex (%)	106 (70.7)	24 (48.0)	60 (66.7)	0.004	0.516
Marriage				0.910	0.963
Married (%)	127 (84.7)	42 (84.0)	76 (84.4)		
Unmarried or others (%)	23 (15.3)	8 (16.0)	14 (15.6)		
Education				0.514	0.578
Illiteracy (%)	29 (19.3)	11 (22.0)	18 (20.0)		
Primary school (%)	53 (35.3)	22 (44.0)	38 (42.2)		
Junior high school (%)	45 (30.0)	10 (20.0)	20 (22.2)		
High school and above (%)	23 (15.3)	7 (14.0)	14 (15.6)		
Previous history					
CVD (%)	36 (24.0)	11 (22.0)	20 (22.2)	0.773	0.753
Diabetes (%)	65 (43.3)	9 (18.0)	28 (31.1)	0.001	0.060
HL (%)	78 (52.0)	21 (42.0)	47 (52.2)	0.221	0.973
Risk factors					
Smoking (%)	61 (40.7)	13 (26.0)	26 (28.9)	0.063	0.066
Drinking (%)	54 (36.0)	6 (12.0)	19 (21.1)	0.001	0.015
SBP (mmHg)	158.56 ± 20.76	143.54 ± 15.65	142.06 ± 18.39	<0.001	<0.001
DBP (mmHg)	88.18 ± 13.23	83.24 ± 10.16	84.46 ± 10.61	0.017	0.024
MAP (mmHg)	111.64 ± 13.32	103.34 ± 10.06	103.66 ± 11.76	<0.001	<0.001
Clinical characteristics					
NIHSS score	2.5 (1, 4)	2 (1, 3)	2 (1, 3)	0.123	
mRS score	2 (1, 3)	1 (0, 2)	1 (0, 2)	0.044	
Laboratory index					
CRP (mg/L)	3.30 (3.13, 6.23)	3.30 (2.98, 3.33)	3.13 (3.11, 3.30)	0.106	0.011
Hs-CRP (mg/L)	1.955 (0.93, 5.88)	0.94 (0.45, 2.77)	1.045 (0.50, 1.83)	0.001	<0.001
FA (ng/mL)	8.20 (5.98, 10.65)	9.60 (7.69, 13.62)	7.85 (5.49, 12.03)	0.006	0.760
VB12 (pg/mL)	291.50 (208.50, 430.76)	415.00 (297.75, 646.50)	330.00 (234.25, 474.00)	0.002	0.069
D-dimer (μg/ml)	0.39 (0.30, 0.68)	0.36 (0.26, 0.68)	0.40 (0.25, 0.62)	0.418	0.322
Troponin (ng/mL)	0.012 (0.012, 0.015)	0.012 (0.012, 0.012)	0.012 (0.012, 0.012)	0.167	0.043
HbA1c (%)	5.98 (5.52, 7.16)	5.79 (5.42, 6.49)	5.91 (5.51, 6.70)	0.231	0.325
FBG (mmol/L)	5.53 (4.82, 6.68)	5.25 (4.78, 6.10)	5.04 (4.60, 6.21)	0.265	0.007
ALT (U/L)	15 (12, 23)	17 (12, 23)	17 (13, 23)	0.679	0.275
AST (U/L)	17 (15, 23)	18.5 (16, 23)	19 (16, 24)	0.452	0.220
UA (μmol/L)	325 (269, 402)	304 (245, 332)	338 (290, 399)	0.007	0.290
Hcy (μmol/L)	12.35 (11.18, 14.80)	8.45 (7.56, 9.20)	12.20 (10.58, 14.82)	<0.001	0.190
TG (mmol/L)	1.535 (1.14, 2.025)	1.43 (0.96, 1.70)	1.61 (1.24, 2.14)	0.044	0.369
TC (mmol/L)	4.32 ± 0.97	4.51 ± 1.00	4.17 ± 1.09	0.230	0.191
HDL (mol/L)	0.93 (0.78, 1.13)	1.05 (0.93, 1.28)	0.99 (0.84, 1.25)	0.001	0.097
LDL (mmol/L)	2.78 ± 0.91	2.95 ± 0.97	2.41 ± 0.89	0.267	0.001
FT3 (pg/mL)	2.88 (2.65, 3.20)	2.94 (2.77, 3.27)	3.01 (2.79, 3.21)	0.074	0.047
FT4 (ng/dL)	1.17 (1.05, 1.30)	1.15 (1.04, 1.24)	1.14 (1.06, 1.26)	0.740	0.572
TSH (μIU/mL)	1.78 (1.09, 2.61)	2.19 (1.36, 3.31)	2.11 (1.37, 2.76)	0.017	0.050

ISHH, H-type hypertension group; NISHH, non-hypertension group; HH, control of H-type hypertension group; CVD, cerebrovascular disease; HL, hyperlipemia; SBP, systolic blood pressure; DBP, diastolic blood pressure; CRP, C-reactive protein; Hs-CRP, hypersensitive C-reactive protein; FA, folic acid; VB12, vitamin B12; HbA1c, glycosylated hemoglobin; FBG, fasting blood glucose; ALT, alanine transaminase; AST, aspartate aminotransferase; UA, uric acid; Hcy, homocysteine; TG, triglyceride; TC, total cholesterol; HDL, high-density lipoprotein; LDL, low-density lipoprotein; FT3, free triiodothyronine; FT4, free tetraiodothyronine; TSH, thyroid stimulating hormone.

**Table 2:** Multivariate logistic regression analysis.

	B (SE)	*p* value	OR	95%CI
HHTN	0.933 (0.448)	0.038^*^	2.542	1.055–6.120
NIHSS score	0.212 (0.067)	0.002^**^	1.236	1.083–1.410
FA	−0.096 (0.04)	0.018^*^	0.908	0.839–0.983

^*^P < 0.05, ^**^P < 0.01.

### 3.2. Diversity and distribution of gut microbiota in three groups

**Figure 1:**
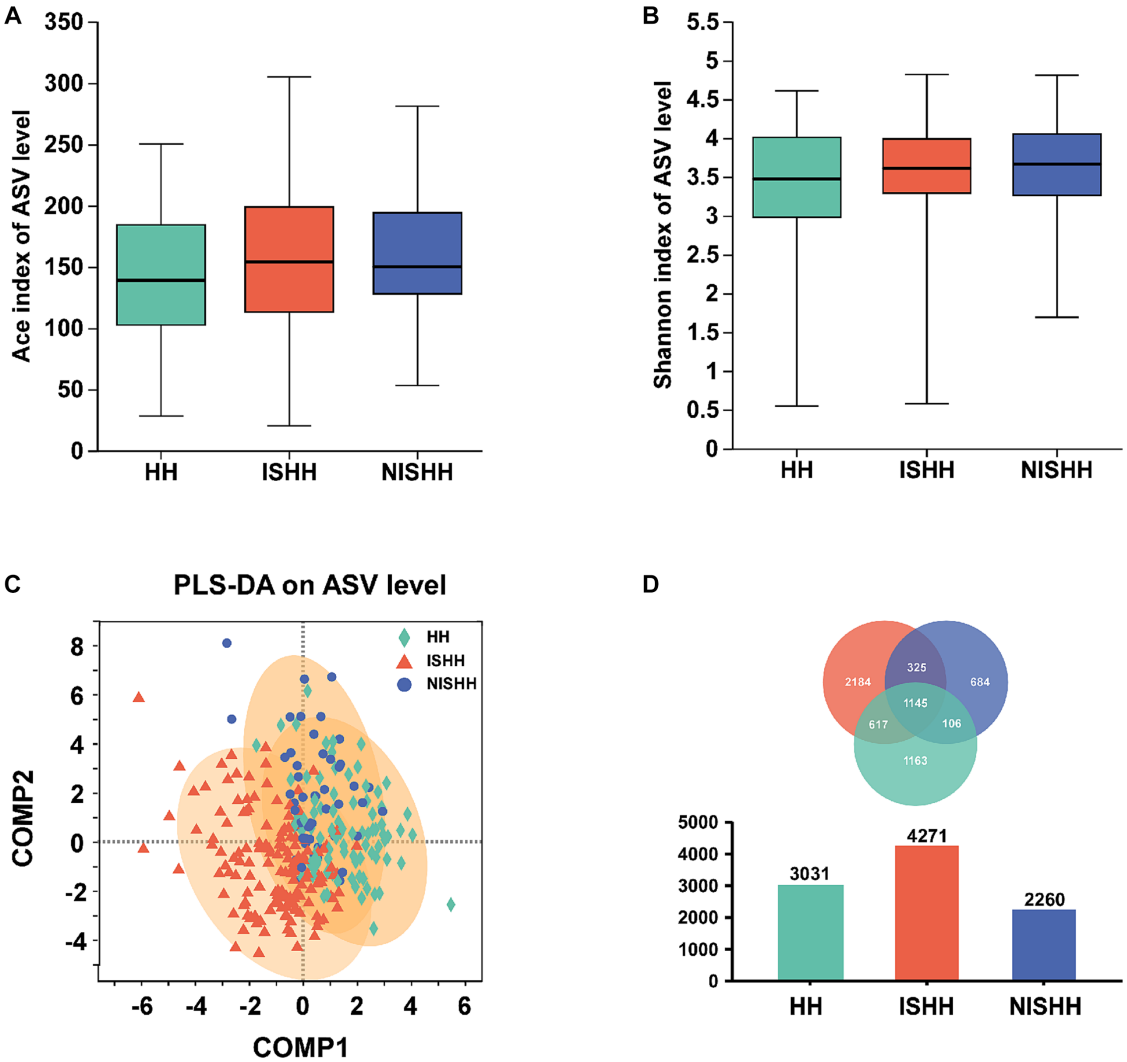
Analysis of gut microbiota among HH, ISHH, and NISHH. **(A,B)** Alpha-diversity analysis in three groups, comparing ACE and Shannon indices changes (Wilcoxon rank-sum test). **(C)** PLS-DA diagram on ASV level. **(D)** A Venn diagram reflected different species and overlaps on the ASV level.

As shown in Figure 1, the ACE (*p* > 0.05, Figure 1A) and Shannon (*p* > 0.05, Figure 1B) indices indicated that alpha diversity had no significant changes in the three groups. As for beta diversity, a comparative analysis of GM composition in various groups was visualized in the form of a PLS-DA diagram, which showed an obvious separation trend in three groups (Figure 1C). The Venn diagram exhibited 3,031 ASVs in the HH group, 4,271 ASVs in the ISHH group, and 2,261 ASVs in the NISHH group (Figure 1D). Meanwhile, 1,145 ASVs were shared in three groups, while the number of unique ASVs was 1,163 in the HH, 2184 in the ISHH, and 684 in the NISHH groups.

**Figure 2:**
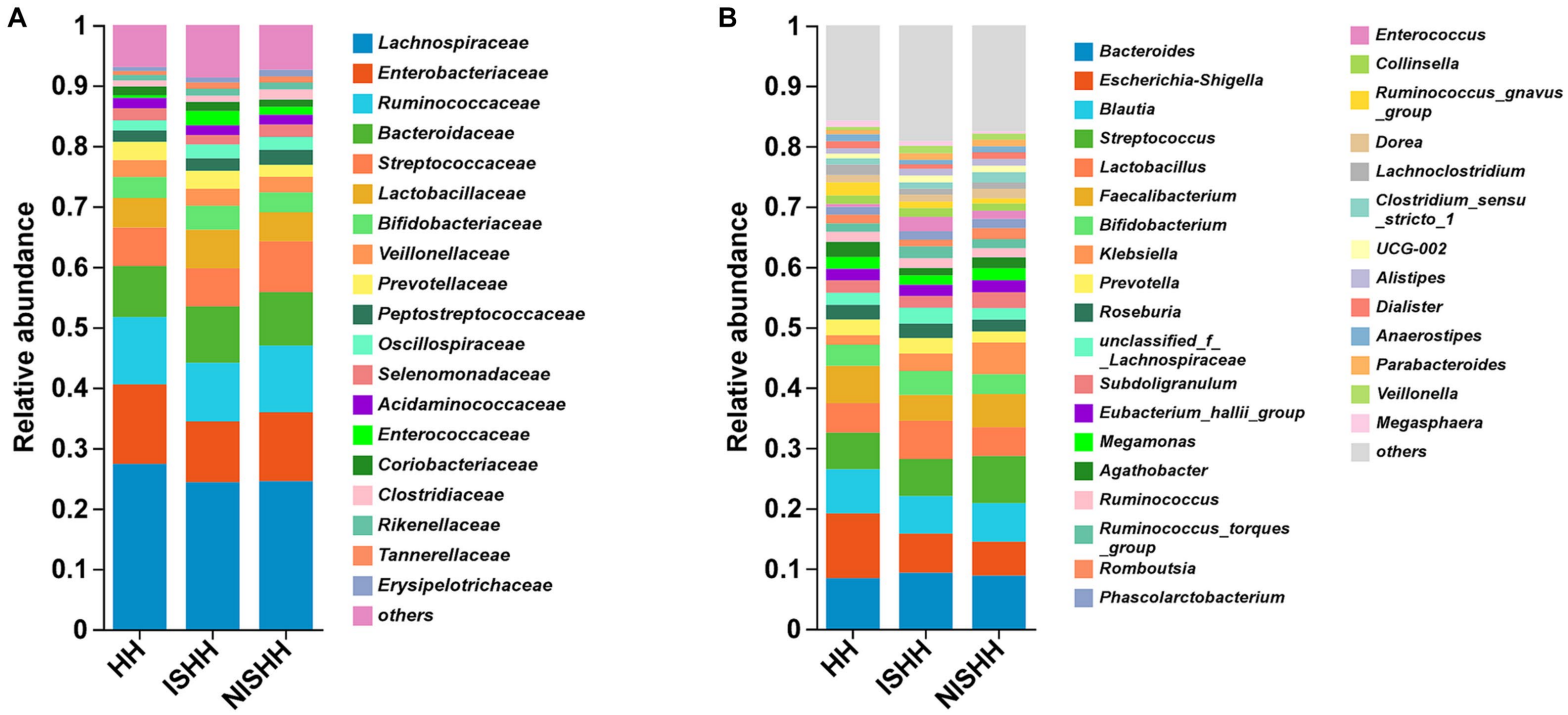
Distribution of gut microbiota among three groups. **(A)** Comparison of different taxa at the family level among three groups. **(B)** Comparison of different taxa at the genus level among three groups.

The histogram of community composition in Figure 2 reflects the different microbial distributions. At the family level (Figure 2A), three groups were mainly composed of *Lachnospiraceae*, *Enterobacteriaceae*, *Ruminococcaceae*, *Bacteroidaceae*, *Streptococcaceae*, *Lactobacillaceae*, *Bifidobacteriaceae*, *Prevotellaceae*, *Veillonellaceae,* and *Peptostreptococcaceae*. However, compared with the NISHH group, the ISHH group was enriched in *Bacteroidaceae*, *Lactobacillaceae, Bifidobacteriaceae,* and *Prevotellaceae*, while the abundance of *Enterobacteriaceae, Ruminococcaceae,* and *Streptococcaceae* decreased. Meanwhile, compared with the HH group, the ISHH group showed that the abundance of *Bacteroidaceae* and *Lactobacillaceae* increased while *Lachnospiraceae*, *Enterobacteriaceae,* and *Ruminococcaceae* were observed to decrease. According to Figure 2B, at the genus level, *Bacteroides* (ISHH: 9.31%, NISHH:8.86%)*, Escherichia-Shigella* (ISHH: 6.46%, NISHH: 5.60%)*, Lactobacillus* (ISHH: 6.38%, NISHH: 4.78%)*, Bifidobacterium* (ISHH: 3.94%, NISHH: 3.29%), and *Prevotella* (ISHH: 2.58%, NISHH: 1.87%) were observed to increase, while bacteria of genera *Streptococcus* (ISHH: 6.08%, NISHH: 7.77%), *Faecalibacterium* (ISHH: 4.28%, NISHH: 5.51%), and *Klebsiella (ISHH: 2.92%, NISHH: 5.22%)* were observed to decrease. At the same time, a comparison between the ISHH group and the HH group indicated that the ISHH group was enriched with *Bacteroides* (ISHH: 9.31%, HH: 8.40%), *Lactobacillus* (ISHH: 6.38%, HH: 4.87%), *Bifidobacterium* (ISHH: 3.94%, HH: 3.46%), and *Klebsiella* (ISHH: 2.92%, HH: 1.61%), while *Escherichia-Shigella* (ISHH: 6.46%, HH: 10.75%), *Blautia* (ISHH: 6.26%, HH: 7.31%), and *Faecalibacterium* (ISHH: 4.28%, HH: 6.22%) were less enriched than the HH group.

### 3.3. Characteristic microbiota between the ISHH group and the NISHH group

**Figure 3:**
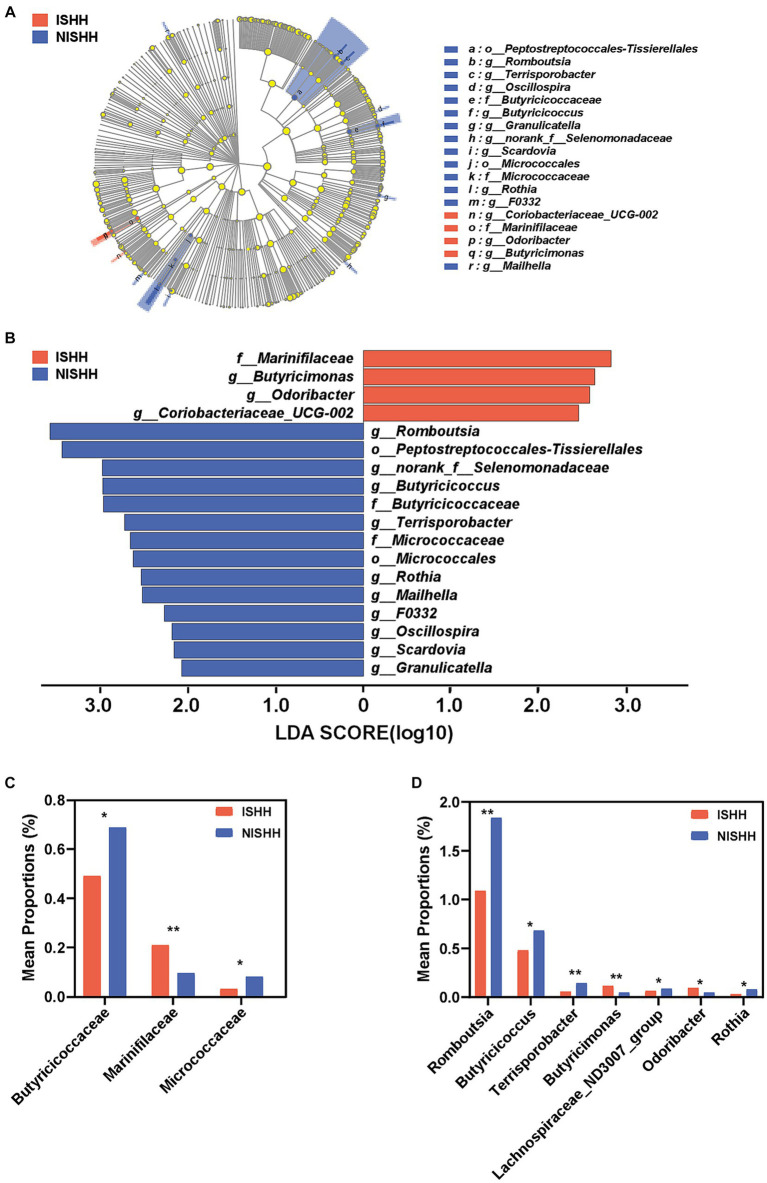
Taxonomic biomarkers of gut microbiota between ISHH and NISHH. **(A)** Cladogram showing the phylogenetic relationships of bacteria taxa. **(B)** LEfSe analysis revealed significant bacterial differences between the ISHH (red) and NISHH (blue) groups. The LDA scores (log10) > 2.0 and a *p*-value <0.05 were listed. **(C)** Histogram of bacterial differences at the family level. **(D)** Histogram of bacterial differences based at the genus level. *p < 0.05, compared with the NISHH group; Error bars indicate SD, **p < 0.01, compared with the NISHH group.

As shown in Figure 3A, the LEfSe cladogram revealed the most significant taxa, representing differences between the two groups from the phylum to genus level. As shown in Figure 3B, 18 taxa with LDA scores of >2 were selected as characteristic microbiota to distinguish between the two groups. Significant bacteria of the ISHH group mainly included *Marinifilaceae*, *Butyricimonas*, *Odoribacter,* and *Coriobacteriaceae_UCG-002*, while *Romboutsia*, *Peptostreptococcales-Tissierellales*, *norank_f__Selenomonadaceae*, *Butyricicoccus*, *Butyricicoccaceae*, *Terrisporobacter*, *Micrococcaceae*, *Micrococcales*, *Rothia*, *Mailhella*, *F0332*, *Oscillospira*, *Scardovia*, *and Granulicatella* were involved in the NISHH group. At the family level, the ISHH group exhibited lower relative abundances of *Butyricicoccaceae* (*p* < 0.05) and *Micrococcaceae* (*p* < 0.05) than the NISHH group, while the abundance of *Marinifilaceae* (*p* < 0.01) was increased (Figure 3C). At the genus level, compared with the NISHH group, in the ISHH group, the relative abundance of *Romboutsia* (*p* < 0.01), *Butyricicoccus* (*p* < 0.05), *Terrisporobacter* (*p* < 0.01), *Lachnospiraceae_ND3007_group* (*p* < 0.05), and *Rothia* (*p* < 0.05) was decreased (Figure 3D), while the relative abundance of *Butyricimonas* (*p* < 0.01) and *Odoribacter* (*p* < 0.05) was increased.

### 3.4. Characteristic microbiota between the ISHH and HH groups

**Figure 4:**
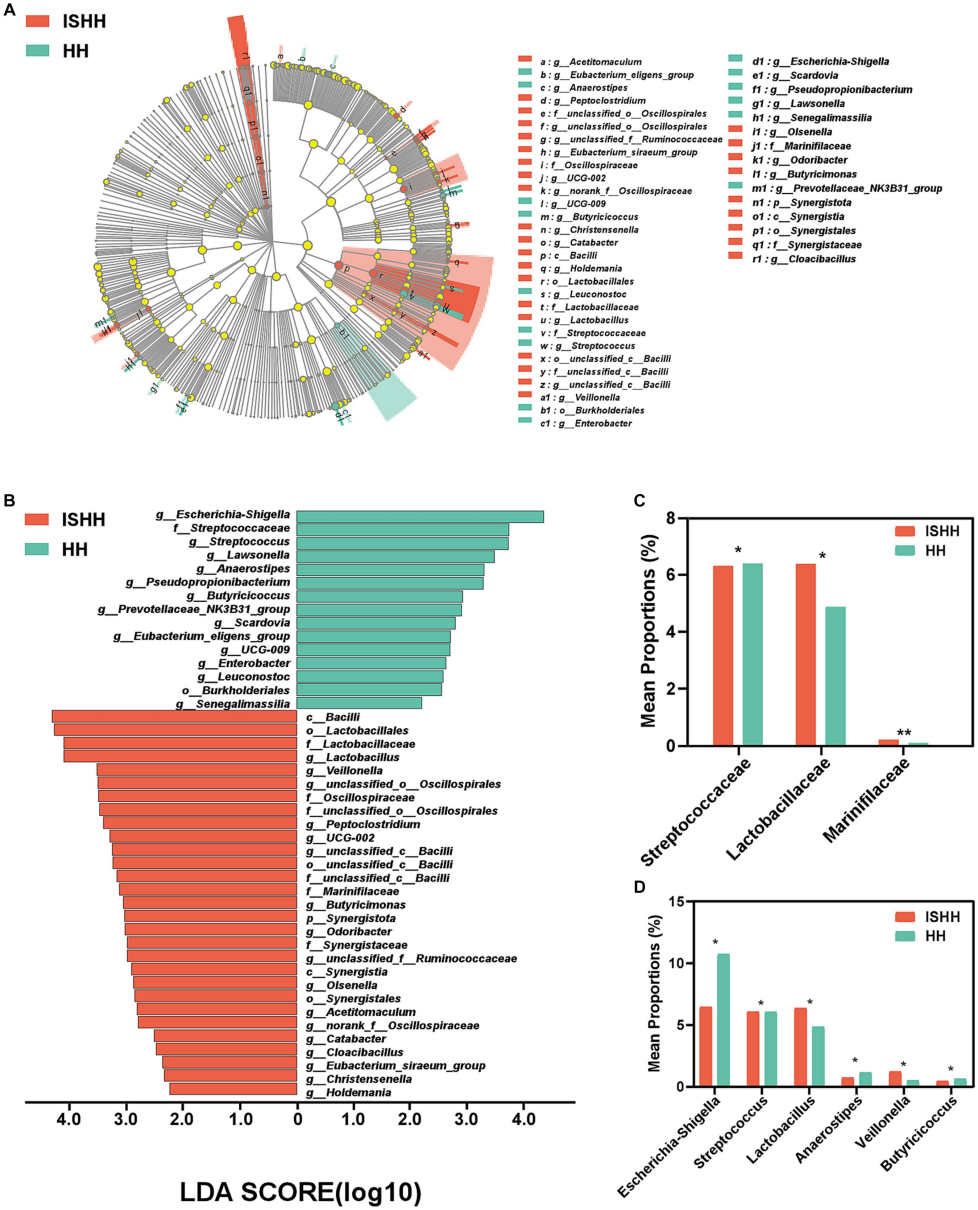
Taxonomic biomarkers of gut microbiota between ISHH and HH. **(A,B)** Cladogram representing the phylogenetic relationships of bacteria taxa and LDA scores between the ISHH and HH groups (LDA scores >2). **(C,D)** Histogram of bacterial differences at the family and the genus levels (Wilcoxon rank-sum test). Error bars indicate SD, *p < 0.05, **p < 0.01.

As shown in Figure 4A, the LEfSe cladogram showed the significant taxa from the phylum to genus levels that could distinguish two groups. As shown in Figure 4B, the LDA bar chart exhibited 44 characteristic taxa (LDA > 2.0). As for differential bacteria at the family level, Figure 4C reflected that the relative abundance of *Streptococcaceae* (*p* < 0.05), *Lactobacillaceae* (*p* < 0.05), and *Marinifilaceae* (*p* < 0.01) were deceased in the HH group compared to the ISHH group. At the genus level (Figure 4D), the relative abundance of *Lactobacillus* (*p* < 0.05) and *Veillonella* (*p* < 0.05) was found to be decreased in the HH group. On the contrary, the relative abundance of *Escherichia-Shigella* (*p* < 0.05), *Streptococcus* (*p* < 0.05), *Anaerostipes* (*p* < 0.05), and *Butyricicoccus* (*p* < 0.05) were increased compared to the ISHH group.

### 3.5. Correlation analysis between differential GM and clinical indicators

**Figure 5:**
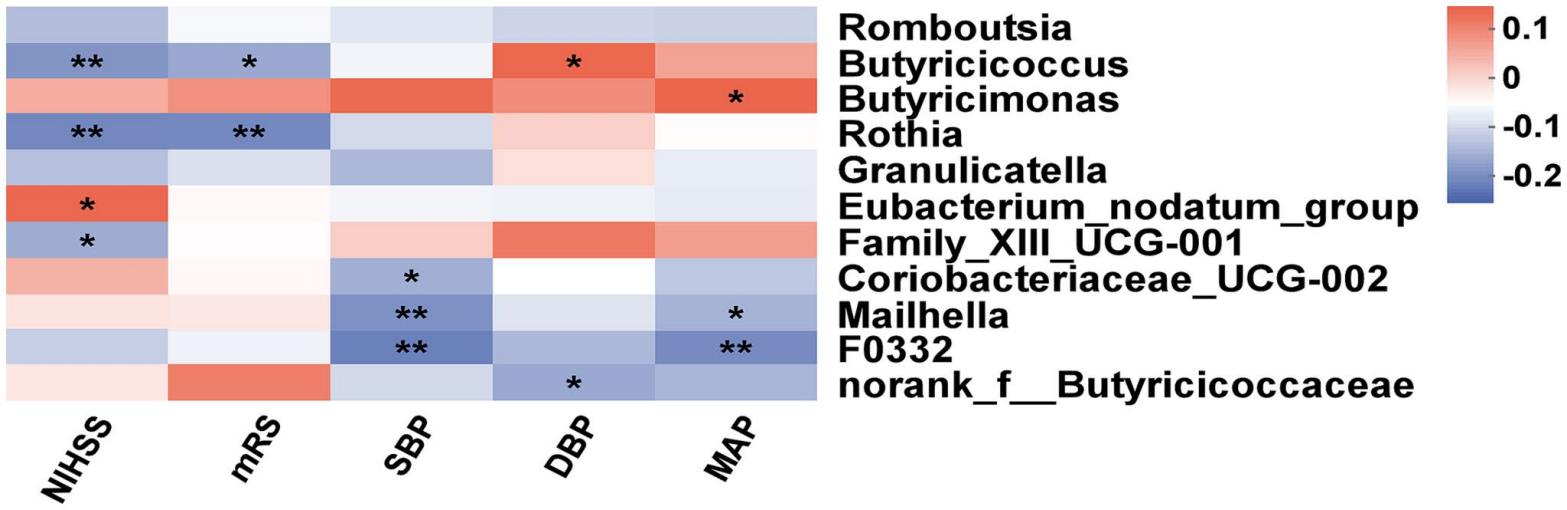
Correlation analysis of gut microbiota with clinical indexes. The heatmap between differential bacteria and clinical factors (NISHH score, mRS score, SBP, DBP, and MAP). The red color was positively correlated, and the blue color was negatively correlated. Deeper colors indicated higher correlation values. *p < 0.05, **p < 0.01.

As shown in Figure 5, the heatmap indicated that the NIHSS score was negatively associated with *Butyricicoccus* (*p* < 0.01), *Rothia* (*p* < 0.01), and *Family_XIII_UCG-001* (*p* < 0.05) and positively associated with *Eubacterium_nodatum_group* (*p* < 0.05). Simultaneously, the abundance of *Butyricicoccus* (*p* < 0.05) and *Rothia* (*p* < 0.01) was negatively associated with the mRS score. There was a negative effect of SBP on genera such as *Coriobacteriaceae_UCG-002* (*p* < 0.05), *Mailhella* (*p* < 0.01), and *F0332* (*p* < 0.01) and a positive effect of DBP on *Butyricicoccusc* (*p* < 0.05), as well as a negative effect of DBP on *norank_f__Butyricicoccaceae* (*p* < 0.05). MAP was negatively associated with the abundance of two genera (*Mailhella* and *F0332*, both *p* < 0.05), while *Butyricimonas* (*p* < 0.01) was positively associated with MAP.

### 3.6. Microbial biomarkers for the prognosis of ISHH patients

**Figure 6:**
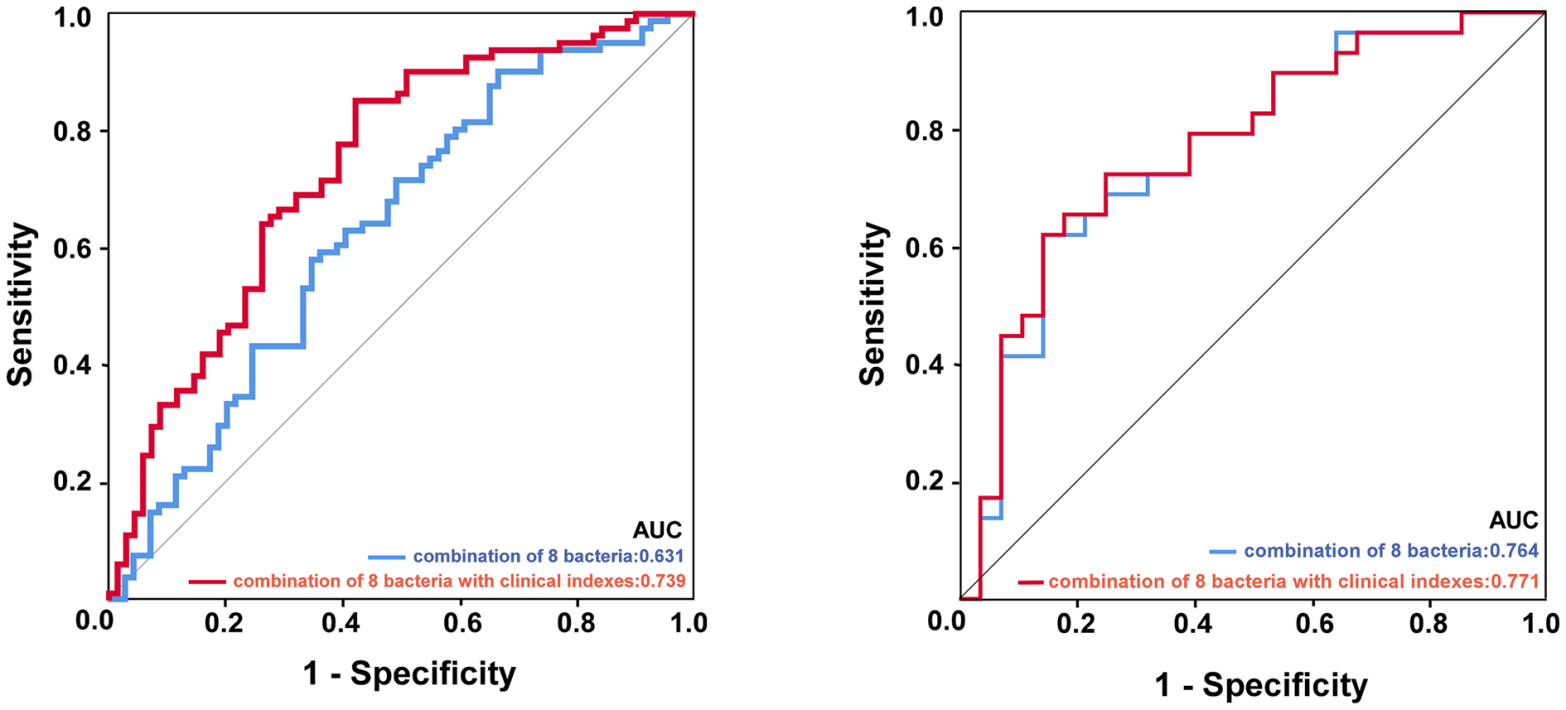
ROC curve analysis of potential microbial biomarkers distinguished the prognosis of ISHH. The image left: experimental cohort, the image right: validation cohort. The blue line represented the combination of eight characteristic bacteria, and the red line represented the combination of eight bacteria with two clinically independent risk factors.

As shown in Figure 6, we screened out eight genera as microbial signatures. *Butyricicoccus*, *Rothia*, *Family_XIII_UCG-001,* and *Eubacterium_nodatum_group* were selected for their correlations with stroke prognostic indexes. In addition to the four genera closely related to the NIHSS and mRS scores, the remaining bacteria came from the top four abundances of different microbiota between the ISHH and NISHH groups, including *Romboutsia, Terrisporobacter, Lachnospiraceae_ND3007_group,* and *Butyricimonas*. The combination of eight bacteria achieved an AUC value of 0.631 (*p* < 0.01, 95% CI: 0.541–0.722) while adding clinically independent risk factors (NIHSS score and low FA) from multivariable logistic regression increased the AUC to 0.739 (*P* < 0.01, 95% CI: 0.659–0.820).

## 4. Discussion

In this study, we revealed the characteristic alteration of GM in ISHH patients from occurrence to outcome. Then, we proposed that bacterial biomarkers, such as composition features and specific bacteria, might participate in the occurrence and development of the disease, further identifying the predictive value of characteristic microbiota.

In this study, an increase in NIHSS score, low FA, and the presence of HHTN were independent risk factors for poor outcomes after stroke onset. The NIHSS score was generally recognized as the tool to evaluate the severity of stroke (31), and a higher score normally indicated more serious neurological deficits (32), which are associated with worse outcomes of stroke (33). In this study, low FA was an independent risk factor for poor outcomes after stroke onset (Table 2), suggesting that supplementation with FA might be associated with a decreased risk of poor prognosis. A recent study showed that an insufficient intake of FA often leads to increased brain damage and a poor prognosis in stroke patients (34). Recent studies have shown that FA administration reduces the risk of stroke by 10–20% (35). FA has potential neuroprotective effects on cerebrovascular diseases (36). It was reported that HHTN participation in aggravating vascular diseases was regarded as a risk factor (11). Our results supported this finding, as the poorer outcome of stroke was performed in the ISHH group, which was specifically manifested by increased mRS scores, resulting in severe disability and a decline in life quality. Another recent report showed that the combination of hypertension and hyperhomocysteinemia played an important role in exacerbating Alzheimer’s disease (AD) and dementia, emphasizing the importance of understanding comorbid vascular risk factors (37). These findings suggested that HHTN, as the combination of hypertension and a high level of Hcy, could negatively affect the development of neurological diseases. However, an explicit mechanism to explain the association between HHTN and AIS remained unclear.

Recent studies have highlighted the influence of gut microbiota on the gut-brain axis and its potential role in stroke. A growing body of evidence suggests that altered microbiota are closely connected with AIS pathogenesis and clinical manifestations (38, 39). In this study, compared with the HH group, there was a significant difference in GM in the ISHH group, which was observed as the increase of proinflammatory bacteria such as *Veillonella* (40), as well as the decrease of genera that could produce SCFAs such as *Butyricicoccus* and *Anaerostipe* (41, 42). Furthermore, the comparison of the ISHH and NISHH groups revealed that decreases in taxa belonging to SCFA-producing bacteria (*Butyricicoccus*, *Romboutsia,* and *Terrisporobacter*) were associated with poor prognosis (43, 44), whereas increases in other bacteria (*Butyricimonas* and *Odoribacter*) were correlated with good outcomes. Interestingly, the decrease of *Butyricicoccus* was observed in the ISHH group compared with the HH and NISHH groups. Thus, we assumed that *Butyricicoccus* might take part in the occurrence and development of stroke in HHTN individuals. *Butyricicoccus*, one of the butyrate-producing genera belonging to *Ruminococcaceae* and Firmicutes (41), was generally considered a beneficial bacterium to human physiology (45, 46). The decrease of *Butyricicoccus* has been observed in various neurological disorders or mental diseases such as PD (47) and multiple sclerosis (48). Previously, we also identified the decrease of *Butyricicoccus* in stroke patients with hyperlipidemia and patients with PSD (24, 29), which reflected the correlations of *Butyricicoccus* with complications of stroke. *Butyricicoccus* was further identified as taking part in immune regulation, enabling it to upregulate expressions of anti-inflammatory factors and to promote neuronal recovery after stroke by activating microglia cells (49). Theo inflammation was responsible for the expansion of infarcts and post-stroke remodeling (50). It was reported that *Butyricicoccus* had the ability to regulate blood pressure in a hypertensive rat model characterized by microbiota dysbiosis and a dysfunctional autonomic nervous system (51). Our results supported this finding, as the relative abundance of *Butyricicoccus* was positively correlated with DBP (Figure 5). Butyrate, the metabolite of *Butyricicoccus*, might be a target for antihypertensive action by regulating the intrarenal renin-angiotensin system (52). In addition to *Butyricicoccus*, we also focused on other characteristic bacteria in the ISHH group. *Rothia* was negatively associated with both the mRS and NIHSS scores, indicating a correlation between decreased *Rothia* and a poor prognosis for the ISHH group. It was reported as an opportunistic pathogen associated with various infectious diseases in immunocompromised individuals (53). Recently, Chang et al. revealed that *Rothia* was enriched in the AIS group with good outcomes, indicating the beneficial function of *Rothia* in stroke (54), which was consistent with our result. *Veillonella* was a kind of proinflammatory taxa as well as *Rothia* and was more abundant in cachectic cancer patients (55). In this study, we assumed that increased *Veillonella* in ISHH patients might be a potential pathogen associated with stroke (Figure 4D). Furthermore, taxa decreased in ISHH patients, such as *Romboutsia*, *Butyricicoccus*, and *Terrisporobacter*, which belonged to the kind of bacteria that could produce SCFAs, which involved acetate, propionate, and butyrate (56). SCFAs were beneficial in modulating hypertension and AIS in animal models (57, 58). It was reported that bacteria producing SCFAs were significantly decreased in female rats with AIS, and these microbial dysbiosis could affect the signal profile to aggravate the stroke through the gut–brain axis (59). SCFAs, especially butyrate, play a vital role in inhibiting inflammation of brain cells or tissue (60). It was reported that butyrate could relieve symptoms of central nervous system diseases by promoting neuronal plasticity, enhancing memory, restoring cognitive damage, and reducing neurotoxicity and neuroinflammation (61–63). Chen et al. confirmed that supplementation with butyrate in model rats with AIS could effectively remodel the GM and intestinal permeability and improve the neurological deficits (64). Another study found that patients with hypertension had a lower abundance of genera that could produce SCFAs (57). SCFAs were also indicated to have the ability to alleviate hypertension, mitigate systemic inflammation, and decrease aortic atherosclerotic lesion area (65), which revealed the important association between SCFA-producing bacteria and vascular diseases (66). In this study, we revealed the alteration of SCFA-producing genera in ISHH, whose decreases were not only observed in the comparison with HH but also in the difference from NISHH. Therefore, decreases in SCFA-producing taxa were supposed to be involved in the pathogenesis of stroke. Gut dysfunction has emerged as a contributor to hypertension, the leading risk factor for stroke. The gut-brain axis has been shown to play a vital role in the prognosis of ISHH, which is associated with gut microbiota changes. In turn, the gut microbiota and its derived metabolites can influence stroke outcomes, and the relationship between gut microbiota, hypertension, and stroke outcomes may be a future research trend.

However, there were several limitations to this study. First, this study was conducted in a single center, so we could not observe the dynamic changes between participants and GM. Second, we still lacked information about microbial metabolites such as SCFAs, which restricted further research associated with GM and ISHH. Finally, the sample amounts for this study were limited. Thus, more patients should be involved in future research. Moreover, lifestyle information, such as lifestyle, exercise, and dietary habits, was not recorded. Despite the limitations of this study, we first described the characteristic GM in ISHH patients, which might be potential biomarkers for apoplectic prognosis.

In conclusion, this study revealed the characteristic bacteria of AIS patients with HHTN and established the model for prognosis based on microbial biomarkers such as *Butyricicoccus*, *Romboutsia*, and *Terrisporobacter*. These findings suggested that key bacteria might participate in the process of ISHH and could act as novel diagnostic biomarkers for prognosis, which might help predict the outcome of AIS patients with HHTN and facilitate the early warning of stroke in HHTN individuals.

## Data availability statement

The datasets presented in this study can be found in online repositories. The names of the repository/repositories and accession number(s) can be found at: https://www.ncbi.nlm.nih.gov/, PRJNA1004022.

## Ethics statement

The studies involving humans were approved by Ethics Committee of the Second Affiliated Hospital of Wenzhou Medical University. The studies were conducted in accordance with the local legislation and institutional requirements. The participants provided their written informed consent to participate in this study.

## Author contributions

SY: Writing – original draft. JC: Data curation, Investigation, Writing – review & editing. YZ: Data curation, Investigation, Writing – review & editing. XL: Software, Writing – review & editing. QC: Investigation, Methodology, Project administration, Writing – review & editing. HX: Formal analysis, Methodology, Writing – review & editing. JL: Investigation, Project administration, Writing – review & editing. JS: Project administration, Writing – review & editing. SZ: Writing – review & editing.
